# Role of Perirectal Fat in the Carcinogenesis and Development of Early-Onset Rectal Cancer

**DOI:** 10.1155/2022/4061142

**Published:** 2022-03-22

**Authors:** An Fu Pan, Nan Xin Zheng, Jin Wang, Jean Luc Tshibangu Kabemba, Kuo Zheng, Fu Shen, Wei Zhang, Xian Hua Gao

**Affiliations:** ^1^Department of Colorectal Surgery, The First Affiliated Hospital, Naval Medical University, Shanghai, China; ^2^Hereditary Colorectal Cancer Center and Genetic Block Center of Familial Cancer, Changhai Hospital, Shanghai, China; ^3^Department of Radiology, The First Affiliated Hospital, Naval Medical University, Shanghai, China; ^4^Department of General Surgery, Central Military Hospital, Kinshasa, Democratic Republic of the Congo

## Abstract

**Purpose:**

The incidence of early-onset rectal cancer (EORC) has been increasing since the past decade, while its underlying cause remained unknown. This study was aimed at clarifying the relationship between perirectal fat area (PFA) and EORC. *Patients and Methods*. All patients with rectal cancer who received radical excision between January 2016 and December 2017 at our hospital were included. The fat series images of pelvic magnetic resonance imaging scans were obtained and PFA at the ischial spine level was calculated using the ImageJ software.

**Results:**

A total of 303 patients were finally included and divided into two groups according to the median PFA: Group 1 (<20.2 cm^2^, *n* = 151) and Group 2 (≥20.2 cm^2^, *n* = 152). PFA positively correlated with body weight and body mass index. PFA increased with invasion depth, lymph node metastasis, TNM stage, tumor deposits, and vascular invasion. Patients with EORC had higher PFA than those with late-onset rectal cancer (LORC; *P* = 0.009). Among patients with stage I–III rectal cancers, those in Group 2 had significantly shorter disease-free survival (*P* = 0.010) and overall survival (*P* = 0.034) than those in Group 1, and PFA was an independent predictor of disease-free survival (OR: 1.683 [1.126-3.015], *P* = 0.035) and overall survival (OR: 1.678 [1.022-2.639], *P* = 0.046).

**Conclusions:**

Patients with EORC had significantly higher PFA than those with LORC. PFA is positively correlated with T stage, N stage, TNM stage, tumor deposit, and vascular invasion and is an independent predictor of disease-free survival and overall survival. Therefore, perirectal fat may be involved in the carcinogenesis and development of EORC.

## 1. Introduction

Colorectal cancer (CRC) is a common malignancy worldwide [1]. Early-onset colorectal cancer (EOCRC) is usually defined as CRC occurring in patients aged <50 years. Since the 1990s, due to the widespread CRC screening in the general population older than 50 years, the incidence and mortality of late-onset colorectal cancer (LOCRC; CRC diagnosed in patients older than 50 years) have significantly decreased [2]. In contrast, the prevalence of EOCRC has rapidly increased in the past decades globally [3–6]. In the USA, it had been reported to rise from 8.6 per 100,000 in 1992 to 13.1 per 100,000 in 2016 [2]. In China, the age-standardized incidence rate of EOCRC increased by 2.76% per year from 1990 to 2016 [1]. Currently, EOCRC is ranked second in cancer prevalence and third in cancer mortality in American people younger than 50 years [7]. It accounts for 10–12% of all newly diagnosed CRC [2]. The incidence of EOCRC is predicted to increase by >140% by 2030 [3, 8], and EOCRC will account for about 11% of colon carcinomas and 23% of rectal carcinomas [2]. A large study with 1,185,763 cases of CRC showed that EOCRC was more likely to be rectal cancer (vs. colon tumors) than LOCRC (40.0% vs. 28.5%, *P* < 0.0001) [9]. Therefore, it is necessary to explore the underlying cause of the increasing trend in early-onset rectal cancer (EORC).

Possible risk factors of EOCRC included global westernization of diets, increased consumption of red meats, stress, antibiotics, synthetic food dyes, sedentary behavior, and gut microbiota [6]. However, the underlying reason for the increasing trend of EOCRC remains unknown [2]. Some scientists had suggested that obesity would contribute to the increasing trend of EOCRC [10–13]. Overweight and weight gain had been reported to be associated with a higher incidence of EOCRC [14, 15]. Accumulation of abdominal fat has a similar effect on the risk of CRC [16]. Traditionally, body mass index (BMI) is the most used parameter for describing overall obesity because it can be easily calculated [17]. Parameters related to abdominal obesity, such as waist circumference, areas of abdominal adipose tissue, and visceral adipose tissue (VAT), have been proven to be more accurate in predicting many cancers types than the overall obesity-related parameters [18]. VAT can be precisely calculated using magnetic resonance imaging/computed tomography (MRI/CT) and can predict CRC incidence, development, and outcomes better than BMI [18–22]. Perirectal fat, a part of the visceral fat surrounding the rectum within the mesorectal fascia (MRF), is speculated to play a part in the progression of rectal cancer by secreting multiple cytokines and adipokines and having direct contact with the rectum. MRI has been widely used in the preoperative evaluation of rectal cancer [23]. MRI outweighs CT in measuring perirectal fat, as adipose tissues have a specific signal on MRI compared to the neighboring tissue [24]. The fat series of pelvic MRI was chosen to measure the perirectal fat area (PFA) because of the aforementioned reasons.

To the best of our knowledge, no studies have explored the role of PFA in the carcinogenesis and development of EORC. Hence, this study was aimed at investigating the impact of PFA on the age at diagnosis of rectal cancer and various tumor development-related parameters, including pathological TNM stage, tumor deposit, vascular invasion, and perineural invasion. In addition, the associations among PFA, body weight, BMI, and oncologic outcomes were also investigated.

## 2. Materials and Methods

### 2.1. Patients

All patients with rectal cancer undergoing radical resection at Changhai Hospital from January 2016 to December 2017 were carefully screened for inclusion in this research. The Colorectal Surgery Department of Changhai Hospital is a tertiary referral center that focuses on CRC treatment. Data of demographic variables, clinicopathological parameters, and oncological outcomes had been prospectively collected and updated in our CRC database. All inpatient, outpatient, operation, postoperative pathological reports, and electronic magnetic resonance (MR) images had been carefully reviewed. This study was approved by the ethics committee of the First Affiliated Hospital, Naval Medical University.

### 2.2. Inclusion Criteria and Exclusion Criteria

Patients were included if they met all of these criteria: (1) aged between 18 and 75 years; (2) rectal cancer within 15 cm from the anal margin under flexible colonoscopy; (3) pathologically confirmed rectal adenocarcinoma; (4) follow-up at our hospital for at least 1 year after the operation date; (5) had pelvic MR images in the picture archiving and communication (PACS) system of our hospital before the operation date; (6) underwent radical excision of rectal cancer in our hospital; and (7) complete clinicopathological data available in our database.

Exclusion criteria included the following: (1) patients with Lynch syndrome or familial adenomatous polyposis; (2) patients with synchronous or metachronous multiple primary CRC; (3) patients with cancers other than adenocarcinoma; (4) patients who only had local excision or biopsy of the rectal cancer; (5) patients who received preoperative treatment, including radiation, chemotherapy, target therapy, and immune therapy; (6) patients who did not have preoperative pelvic or rectal MR images in our PACS; (7) preoperative pelvic MR images were too obscure to measure PFA; and (8) preoperative pelvic MR images did not include fat series (DIXON-Fat, LAVA-Flex-Fat, or T1-Fat).

### 2.3. Follow-Up

Postoperative follow-up included digital rectal examination, chest X-ray or CT scan, liver ultrasound or contrast-enhanced MRI, pelvic-enhanced MRI, serum carcinoembryonic antigen, and carbohydrate antigen 19-9 levels. These examinations were carried out every 3 months for the first 2 years postoperatively, every 6 months for the following 3 years, and annually thereafter. Complete colonoscopy was performed 1 year after the surgery and every 1–3 years thereafter.

### 2.4. Measurement of PFA

**Figure 1:**
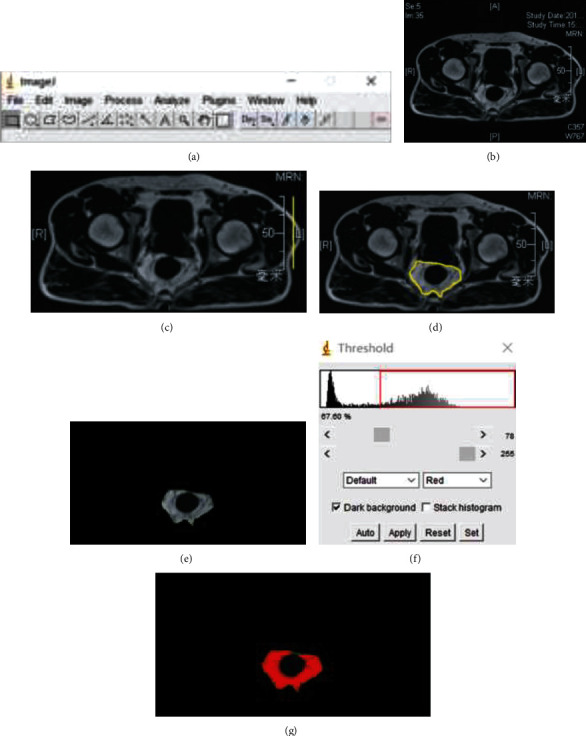
Measurement process of the perirectal fat area (PFA) with the ImageJ software. (a) ImageJ software; (b) the transverse pelvic magnetic resonance image is obtained at the ischial spine level; (c) the scale is set based on the attached ruler; (d) PFA is circled by hand; (e) the outer unrelated region is removed; (f) selecting of the automatic threshold; and (g) the red region demonstrates PFA.

The fat series (DIXON-Fat, LAVA-Flex-Fat, or T1-Fat) images of pelvic contrast MRI scans were obtained from the PACS. PFA was calculated at the ischial spine level. The fat area was measured using the ImageJ software (Figure 1(a)). First, a transverse pelvic MR image was obtained at the ischial spine level (Figure 1(b)). The scale was set based on the attached ruler in the image (Figure 1(c)). Subsequently, the region of interest (PFA) was circled by hand (Figure 1(d)). Only mesorectal fat within the MRF (inside the pelvic wall muscle) was included. The outer unrelated region was removed (Figure 1(e)). The picture was transformed to an 8-bit image, and the automatic threshold was selected [24] (Figure 1(f)). The red region indicates the PFA (Figure 1(g)). Finally, the menu tool “analyze particles” was used to measure the area of the red region [25].

All measurements were performed independently by two physicians (AFP and NXZ), who were blinded to the clinicopathological parameters. Every result was compared between them. If the difference between two results was within 5%, the mean value was chosen as the final result; otherwise, discrepancies were resolved by discussion between the physicians, and with a senior radiologist after their discussion.

### 2.5. Statistical Analysis

All analyses were performed with the SPSS 22.0. The independent sample *t*-test, Mann–Whitney *U* test, and one-way analysis of variance were used in the comparative analysis of quantitative parameters. The Chi-square test and Fisher's exact test were used in the comparative analysis of qualitative parameters. The relationships between PFA, body weight, and BMI were analyzed using Pearson's correlation coefficient. The role of PFA on OS and DFS was analyzed using the Kaplan–Meier survival analysis. Multivariate Cox analyses were selected to identify predictive parameters for OS and DFS. All parameters with statistical and clinical significance in the univariate analysis were included in the following multivariate Cox analysis. Multivariate analyses were performed with the forward stepwise method (likelihood ratio); the inclusion criterion was “*P* < 0.05,” and the exclusion criterion was “*P* > 0.10.” *P* value < 0.05 was considered statistically significant.

## 3. Results

Of the 1,418 patients with rectal cancer who underwent surgery between January 2016 and December 2017 at our hospital, 303 (21.4%) were finally included in the study after excluding patients who had preoperative chemoradiotherapy (*n* = 420) or local excision (*n* = 23), other tumor types (*n* = 12), no pelvic MR image (*n* = 241), obscure MR images (*n* = 11), or no fat series image (*n* = 408). A flowchart of patient screening is shown in Figure S1.

### 3.1. Clinicopathological Features

The median and interquartile range of age at diagnosis and follow-up period were 62 (53–67) years and 48 (42–54) months, respectively. At diagnosis, the mean and standard deviation of body weight, BMI, and PFA were 64.4 ± 10.1 kg, 23.3 ± 2.9 kg/m^2^, and 20.4 ± 6.9 cm^2^, respectively. The 303 patients were classified into two groups using the median of PFA (20.2 cm^2^): Group 1 (<20.2 cm^2^) and Group 2 (≥20.2 cm^2^).

### 3.2. Comparison of Quantitative Variables between Patients with Lower and Higher PFA

Comparisons of quantitative variables between patients with lower PFA (Group 1) and higher PFA (Group 2) are shown in Table 1. Compared to Group 1, Group 2 had significantly younger age and higher height, body weight, BMI at diagnosis, and more retrieved lymph nodes (Table 1).

### 3.3. Comparison of Qualitative Variables between Patients with Lower and Higher PFA

Comparisons of qualitative variables between patients with lower PFA (Group 1) and higher PFA (Group 2) are shown in Table 2. Compared to Group 1, Group 2 had significantly more male patients, lymph node metastasis, tumor deposits, and higher TNM stage (Table 2, all *P* < 0.05).

### 3.4. Relationship between Body Weight, BMI, and PFA

**Figure 2:**
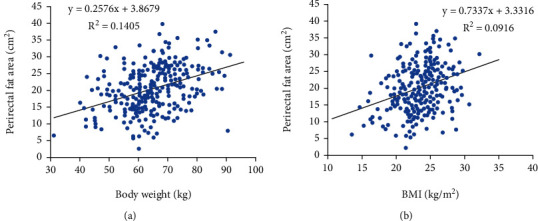
Relationship between body weight, BMI, and PFA.

Pearson's correlation analysis showed that PFA was positively correlated with body weight (*r* = 0.375, *P* < 0.001) and BMI (*r* = 0.302, *P* < 0.001) (Figure 2).

### 3.5. Relationship between PFA, TNM Stage, Tumor Deposit, Vascular Invasion, Perineural Invasion, and Age at Diagnosis of Rectal Cancer

**Figure 3:**
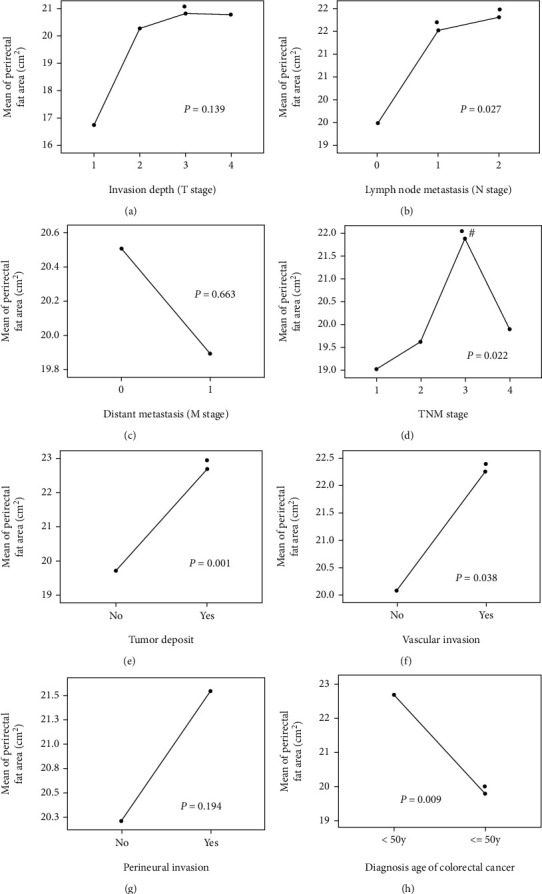
Relationship between perirectal fat area, TNM stage, tumor deposit, vascular invasion, perineural invasion, and age at diagnosis of rectal cancer. ∗Compared with the first group, *P* < 0.05. ^#^Compared with the second group, *P* < 0.05.

One-way analysis of variance showed that PFA increased with T stage; patients with T3 stage had significantly higher PFA than patients with T1 stage (Figure 3(a), *P* < 0.05). Similarly, PFA increased with the N stage (Figure 3(b), *P* = 0.027); patients with N1 and N2 stage had significantly higher PFA than patients with N0 stage (Figure 3(b), both *P* < 0.05). Patients with distant metastasis had slightly lower PFAs than those without distant metastasis; however, there was no statistical significance (Figure 3(c), *P* = 0.663). For the TNM stage, PFA increased from stage I to III but then decreased from stage III to IV (Figure 3(d), *P* = 0.022). Patients with stage III tumor had higher PFA than those with stage I and II (Figure 3(d), both *P* < 0.05). Patients with tumor deposits had higher PFAs than those without (Figure 3(e), *P* = 0.001). Patients with vascular invasion had higher PFAs than those without (Figure 3(f), *P* = 0.038). Patients with perineural invasion had similar PFAs than those without (Figure 3(g), *P* = 0.194). Patients with EORC had higher PFAs than those with LORC (Figure 3(h), *P* = 0.009).

### 3.6. Comparison of DFS and OS between Patients with Lower and Higher PFA

**Figure 4:**
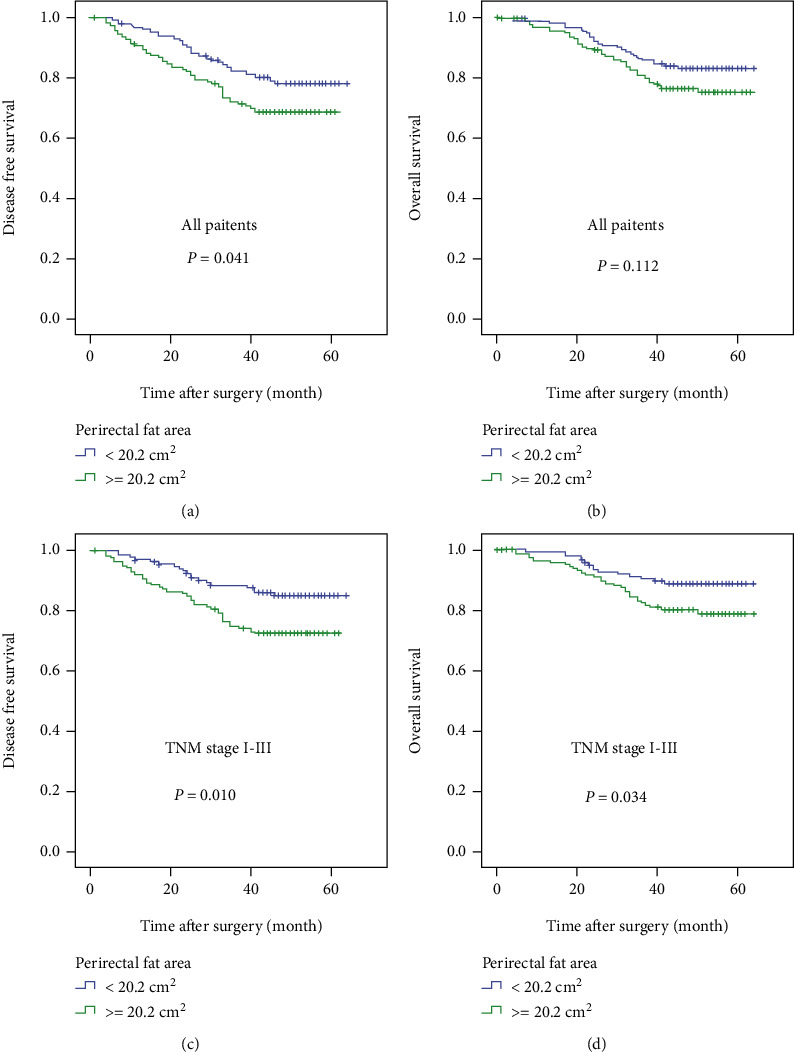
The impact of PFA on DFS and OS among all patients with rectal cancer and those with stage I–III rectal cancer.

A total of 303 patients were classified into two groups based on the median of PFA: Group 1 (<20.2 cm^2^, *n* = 151) and Group 2 (≥20.2 cm^2^, *n* = 152). When all patients were analyzed, Group 2 had significantly shorter DFS (Figure 4(a), *P* = 0.041) and similar OS (Figure 4(b), *P* = 0.112) compared to Group 1. Patients with distant metastasis usually become thinner due to massive nutritional consumption by the tumor, which may lead to a decrease in PFA and interfere with the correlation analysis between PFA and survival duration. After excluding patients with distant metastasis (stage IV), Group 2 had significantly shorter DFS (Figure 4(c), *P* = 0.010) and OS (Figure 4(d), *P* = 0.034) than Group 1.

### 3.7. Univariate and Multivariate Cox Analyses of Potential Predictors of DFS in Patients with Stage I–III Rectal Cancer

The results of the univariate analysis showed that PFA, diabetes, tumor deposit, vascular invasion, invasion depth, and TNM stage were potential predictors of DFS (Table S1). Multivariate analysis showed that PFA [OR: 1.683 (1.126-3.015), *P* = 0.035], vascular invasion, and TNM stage were independent predictors of DFS (Table 3).

### 3.8. Univariate and Multivariate Cox Analyses of Potential Predictors of OS in Patients with Stage I–III Rectal Cancer

The results of the univariate analysis showed that PFA, tumor deposit, vascular invasion, invasion depth, and TNM stage were potential predictors of OS (Table S2). Multivariate analysis showed that PFA [OR: 1.678 (1.022–2.639), *P* = 0.046], vascular invasion, and TNM stage were independent predictors of OS (Table 4).

## 4. Discussion

Our study demonstrated that patients with PFA ≥ 20.2 cm^2^ had significantly younger age at diagnosis; higher body weight, BMI, and TNM stage; and more lymph node metastasis and tumor deposits than patients with PFA < 20.2 cm^2^. PFA positively correlated with body weight and BMI. PFA increased with T stage, N stage, TNM stage, and the presence of tumor deposits and vascular invasion. Patients with EORC had a greater PFA than those with LORC. Univariate and multivariate analyses showed that PFA was an independent predictor of DFS and OS in patients with stage I–III rectal cancer. Our results indicate that perirectal fat might be involved in the carcinogenesis and development of EORC.

A large prospective study with 85,256 healthy women and 114 cases of EOCRC showed that obese women had higher incidence of EOCRC [14]. People with higher BMI at 18 years of age had a higher incidence of EOCRC and weight gain, as the patient aged 18 years had a similar effect [14]. Another large study with 583,511 participants and 3,173 cases of CRC proved that BMI ≥ 30.0 kg/m^2^ contributed to higher incidence of EOCRC [26]. Obesity was proven to play a key role in CRC pathogenesis [27]. Multiple meta-analyses had demonstrated a consistently positive association between obesity and the incidence of CRC [10, 28–30]. Obesity can be classified into overall obesity (body weight and BMI) and abdominal obesity (waist circumference, VAT, and PFA). Abdominal obesity has been reported to be a more sensitive predictor of metabolic diseases and cancers than overall obesity [18]. Both overall obesity and abdominal obesity positively correlated with CRC incidence [27, 30], and the abdominal obesity was more sensitive than overall obesity [29]. A Korean population-based cohort study, which included 9,959,605 participants and 101,197 cases of CRC, demonstrated that a higher incidence of CRC and rectal cancer was associated with abdominal obesity, and these associations were independent of BMI [31]. In recent years, adipose tissue has proven to be the largest human endocrine organ [32]. Different from subcutaneous adipose tissue, VAT could secrete multiple cytokines and adipokines, which could promote CRC carcinogenesis and development [33–35]. Patients with higher VAT had shorter OS and DFS than those with lower VAT [36, 37]. Furthermore, VAT is a more reliable and accurate indicator than BMI in predicting the oncological outcomes of rectal cancer [38, 39].

PFA, as the closest part of the VAT surrounding the rectum, was speculated to play an active role in the carcinogenesis and progression of rectal cancer. Tripathi et al. reported that the mean PFA in a Chinese Han population, measured using pelvic MR images, was 24.0 ± 6.9 cm [23], which was higher than the mean PFA (20.4 ± 6.9 cm^2^) in our study. This may have been caused by the difference in the series of MR images selected for measurement; they selected T2-weighted images, and we selected the fat series images. The fat series images are more specific and accurate for measuring PFA, and some areas of nonfat tissue were excluded, leading to lower PFA in our study. It was reported that viscerally obese patients with rectal cancer were more likely to be male [37], which was consistent with our results. Two other studies measured PFA using pelvic CT/MR images and found that PFA positively correlated with BMI [23, 40], which was consistent with our results. Both BMI and VAT were prognostic factors for OS and DFS in CRC patients [17, 41, 42]. Yoon et al. found that patients with PFA ≥ 10 cm^2^ had significantly longer DFS than those with PFA < 10 cm^2^, and the underlying reason remains unknown [40]. A possible hypothesis raised by the authors was that a higher PFA might increase the probability of negative resection margins. A higher PFA leads to a larger capacity for tumor cells within the MRF and then reduces the incidence of a positive circumferential resection margin [40]. However, in our study, no significant relationship was identified between PFA and the resection margin. Our study showed that in all patients, those with higher PFA had shorter DFS and similar OS than those with lower PFA. In patients with stage I*–*III rectal cancers, those with higher PFA had shorter DFS and OS than those with lower PFA. Patients with distant metastasis have lower PFA due to massive nutritional consumption by the tumor, and shorter DFS and OS; this might lead to contradictory results. In patients with stage IV cancers, lower PFA is associated with shorter survival, while in patients with stage I–III rectal cancers, higher PFA is associated with shorter survival. Some researchers found that low VAT was a poor prognostic marker because it could lead to impairment in nutritional supply and subsequent malnutrition [43, 44]. In addition, higher PFA might affect the surgical difficulty of TME and intersphincteric resection, leading to poor oncological outcomes [45]. This was consistent with the results of our survival analysis.

Our study and the published literature provide strong and consistent evidence that obesity is involved in CRC carcinogenesis and progression. With the development in economy and alteration in lifestyle, more people are increasingly overweight or obese [46], with 16% of children being overweight [47]. Obesity is usually measured by BMI, but the new measurement, PFA, is a more accurate predictor of CRC than BMI. Our study demonstrates that higher PFA is associated with carcinogenesis and development of EORC, and PFA may also play a crucial role in treatment sensitivity and oncological results of EORC. Measuring PFA could help us identify young individuals who might benefit from early screening and specialized surveillance for EORC. Furthermore, it could help predict the survival of patients with rectal cancer [27]. Although our evidence proves that PFA is associated with the incidence, carcinogenesis, and development of EORC, the underlying molecular mechanisms have not been completely clarified [27]. The following factors have been reported to play a vital role in the carcinogenesis and progression of EORC [27, 46, 48]: obesity-induced insulin resistance, chronic inflammation, microbiota, altered levels of adipokines, cytokines, various growth factors, adiponectin, and leptin. Clarifying the molecular mechanisms of PFA on EORC risk is an important strategy for preventing the increasing trend of EORC. Multiple studies had proven that microbiota dysbiosis plays a vital role in obesity, tumorigenesis, growth, immunity dysregulation, diagnosis, and chemotherapy sensitivity in CRC and EOCRC [49, 50]. The administration of probiotics may help microbiota to recover and reduce the incidence of obesity and CRC [49]. Diet could influence the intestinal microbiota and regulate the carcinogenesis of CRC [51]. It has been recently reported that supplementation of allium vegetables and allium-containing food could reduce the formation of preneoplastic colorectal lesions (aberrant crypt foci and adenomatous polyp) by modifying the gut microbiota and reducing the incidence and mortality of CRC [52]. Taken together, probiotics and allium vegetables may be used to modify the intestinal microbiota, decrease the risk of obesity, and thus reduce PFA and the risk of EORC. Further research is required to explore the underlying biological mechanisms regulating the relationship between PFA and EORC with the goal of unraveling a strategy to prevent EORC [27].

Our study has several limitations. First, selection bias was inevitable, as it was a retrospective study and only 21.4% (303/1418) of all patients with rectal cancer were included. This should be considered when interpreting the results. Second, our department is a tertiary referral center specialized in CRC treatment; hence, this might have resulted in more standard surgical procedures, lower incidence of positive resection margins, and better oncological results. Third, the relationship between obesity and EOCRC might vary in different sites of the large intestine [28, 29]. However, we only included patients with rectal cancer in this study. Another study involving the pericolon fat area is required to determine if the same effect exists in colonic cancer. Fourth, VAT was usually measured at the level of the third lumbar spine vertebra, which was not routinely covered by the pelvic MRI scan; therefore, we could not measure VAT using pelvic MR images and compare the role of PFA with VAT.

In conclusion, we described a more accurate method for measuring PFA using fat series images from pelvic MRI scans. Our study indicates that PFA is associated with early age at diagnosis, adverse clinicopathological parameters, and poor oncological outcomes in stage I–III rectal cancer. Therefore, perirectal fat may be involved in the carcinogenesis and development of EORC. Future large prospective studies are required to verify the conclusions drawn from this study.

## Figures and Tables

**Table 1:** Comparison of quantitative variables between patients with lower and higher PFA.

Clinicopathological variables	Perirectal fat area (cm^2^)		*P*
	Group 1 (<20.2, *n* = 151)	Group 2 (≥20.2, *n* = 152)	
Age (year)	61.66 ± 10.36	58.91 ± 10.34	0.022
Height (cm)	164.25 ± 6.86	167.36 ± 7.03	<0.001
Body weight (kg)	61.21 ± 9.64	67.59 ± 9.49	<0.001
Body mass index (kg/m^2^)	22.63 ± 3.03	24.05 ± 2.48	<0.001
Carcinoembryonic antigen (ng/mL)	3.53 (2.09–7.19)	3.25 (1.53–7.73)	0.439
Carbohydrate antigen 19-9 (U/mL)	6.97 (3.2–13.99)	6.41 (3.08–16.36)	0.805
Length of hospital stay (d)	11.93 ± 5.42	11.38 ± 4.95	0.361
Length of postoperative hospital stay (d)	8.33 ± 4.8	7.67 ± 4.62	0.223
Tumor diameter (cm)	3.75 ± 1.45	3.9 ± 1.75	0.400
Positive lymph node	1.46 ± 2.95	1.68 ± 2.78	0.503
Total lymph node	14.07 ± 4.03	15.11 ± 3.86	0.023

**Table 2:** Comparison of qualitative variables between patients with lower and higher PFA.

Clinicopathological variables		Group 1 (PFA <20.2 cm^2^, *n* = 151)	Group 2 (PFA ≥ 20.2 cm^2^, *n* = 152)	*P*
Sex	Male	91 (60.3%)	113 (74.3%)	0.009
	Female	60 (39.7%)	39 (25.7%)	
Surgical procedure	Dixon	134 (88.7%)	139 (91.4%)	0.593
	Miles	15 (9.9%)	10 (6.6%)	
	Hartmann	2 (1.3%)	3 (2%)	
Combined resection	No	143 (94.7%)	146 (96.1%)	0.576
	Yes	8 (5.3%)	6 (3.9%)	
Stoma	No	51 (33.8%)	54 (35.5%)	0.749
	Yes	100 (66.2%)	98 (64.5%)	
Radical resection	No	4 (2.6%)	4 (2.6%)	1.000
	Yes	147 (97.4%)	148 (97.4%)	
Laparoscopic surgery	Open	129 (85.4%)	123 (80.9%)	0.294
	Laparoscopic	22 (14.6%)	29 (19.1%)	
History of other cancer	No	147 (97.4%)	150 (98.7%)	0.448
	Yes	4 (2.6%)	2 (1.3%)	
Family history of cancer	No	137 (90.7%)	136 (89.5%)	0.715
	Yes	14 (9.3%)	16 (10.5%)	
Diabetes	No	133 (88.1%)	138 (90.8%)	0.443
	Yes	18 (11.9%)	14 (9.2%)	
Hypertension	No	99 (65.6%)	100 (65.8%)	0.967
	Yes	52 (34.4%)	52 (34.2%)	
History of appendectomy	No	131 (86.8%)	139 (91.4%)	0.190
	Yes	20 (13.2%)	13 (8.6%)	
Concomitant polyp	No	137 (90.7%)	133 (87.5%)	0.367
	Yes	14 (9.3%)	19 (12.5%)	
Gross appearance	Ulcerative	103 (68.2%)	113 (74.3%)	0.238
	Protruding	48 (31.8%)	39 (25.7%)	
Differentiation	Moderate	123 (81.5%)	126 (82.9%)	0.744
	Poor	28 (18.5%)	26 (17.1%)	
Invasion depth (T stage)	1	13 (8.6%)	4 (2.6%)	0.077
	2	38 (25.2%)	33 (21.7%)	
	3	95 (62.9%)	106 (69.7%)	
	4	5 (3.3%)	9 (5.9%)	
Lymph node metastasis (N stage)	0	94 (62.3%)	72 (47.4%)	0.029
	1	35 (23.2%)	53 (34.9%)	
	2	22 (14.6%)	27 (17.8%)	
Distant metastasis (M stage)	No	137 (90.7%)	140 (92.1%)	0.669
	Yes	14 (9.3%)	12 (7.9%)	
TNM stage	1	36 (23.8%)	24 (15.8%)	0.013
	2	54 (35.8%)	41 (27%)	
	3	47 (31.1%)	75 (49.3%)	
	4	14 (9.3%)	12 (7.9%)	
BRAF	Wild	146 (96.7%)	151 (99.3%)	0.121
	Mutant	5 (3.3%)	1 (0.7%)	
KRAS	Wild	82 (54.3%)	88 (57.9%)	0.529
	Mutant	69 (45.7%)	64 (42.1%)	
Tumor deposit	No	126 (83.4%)	102 (67.1%)	0.001
	Yes	25 (16.6%)	50 (32.9%)	
Vascular invasion	No	130 (86.1%)	122 (80.3%)	0.175
	Yes	21 (13.9%)	30 (19.7%)	
Perineural invasion	No	124 (82.1%)	123 (80.9%)	0.788
	Yes	27 (17.9%)	29 (19.1%)	
Circumferential resection margin	Negative	147 (97.4%)	148 (97.4%)	1.000
	Positive	4 (2.6%)	4 (2.6%)	
Distal resection margin	Negative	150 (99.3%)	150 (98.7%)	1.000
	Positive	1 (0.7%)	2 (1.3%)	
Postoperative chemotherapy	No	43 (28.5%)	44 (28.9%)	0.928
	Yes	108 (71.5%)	108 (71.1%)	
Postoperative radiation	No	117 (77.5%)	123 (80.9%)	0.461
	Yes	34 (22.5%)	29 (19.1%)	

**Table 3:** Multivariate analysis for potential predictors of DFS in patients with stage I–III rectal cancer.

Parameters	OR (95% CI)	*P*
Perirectal fat area (≥20.2 cm^2^ vs. <20.2 cm^2^)	1.683 (1.126–3.015)	0.035
Diabetes (yes vs. no)	1.876 (0.91–3.865)	0.088
Tumor deposit (yes vs. no)	0.515 (0.25–1.06)	0.072
Vascular invasion (yes vs. no)	2.695 (1.529–4.752)	0.001
Invasion depth (T3-4 vs. T1-2)	2.095 (0.96–4.572)	0.063
TNM stage (3-4 vs. 1-2)	3.222 (1.725–6.018)	<0.001

**Table 4:** Multivariate analysis of potential predictors of OS in patients with stage I–III rectal cancer.

Parameters	OR (95% CI)	*P*
Perirectal fat area (≥20.2 cm^2^ vs. <20.2 cm^2^)	1.678 (1.022–2.639)	0.046
Tumor deposit (yes vs. no)	0.834 (0.385–1.808)	0.646
Vascular invasion (yes vs. no)	2.981 (1.595–5.571)	0.001
Invasion depth (T3-4 vs. T1-2)	1.629 (0.693–3.827)	0.263
TNM stage (3-4 vs. 1-2)	4.192 (1.948–9.021)	<0.001

## Data Availability

The data used to support this study are included within this article.

## Supplementary Materials

Supplementary 1 Figure S1: flow chart of patient selection.

Supplementary 2 Table S1: univariate analysis for possible risk factors of DFS in patients with stage I–III CRC.

Supplementary 3 Table S2: univariate analysis for possible risk factors of OS in patients with stage I–III CRC.
