# Supplementary figures and images for: Role of Perirectal Fat in the Carcinogenesis and Development of Early-Onset Rectal Cancer
Source: J Oncol. 2022 Mar 22;2022:4061142. doi: 10.1155/2022/4061142 (PMC8965599; doi:10.1155/2022/4061142)

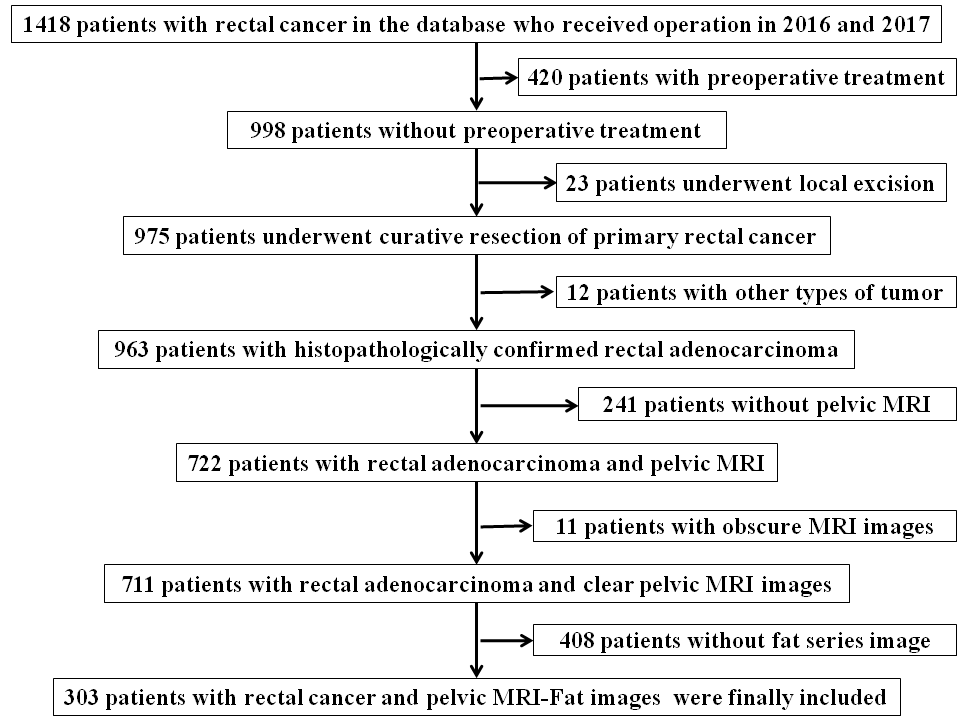


**Figure S1**. Flow chart of patient selection.

Supplement: Supplementary 1 — Figure S1: flow chart of patient selection. [file 4061142.f1.docx]
