# Supplementary material for: Role of Perirectal Fat in the Carcinogenesis and Development of Early-Onset Rectal Cancer
Source: J Oncol. 2022 Mar 22;2022:4061142. doi: 10.1155/2022/4061142 (PMC8965599; doi:10.1155/2022/4061142)
Supplement: Supplementary 2 — Table S1: univariate analysis for possible risk factors of DFS in patients with stage I–III CRC. [file 4061142.f2.docx]

**Table S1.** Univariate analysis for possible risk factors of DFS in patients with stage I–III CRC

| **Parameters** | **Odds ratio (95% CI)** | ***P*** |
| --- | --- | --- |
| Perirectal fat area (≥20.2 cm^2^ vs. <20.2 cm^2^) | 2.029(1.169-3.519) | **0.012** |
| Sex (Female vs. Male) | 0.942(0.539-1.648) | 0.835 |
| Age2 (≥50 y vs. <50 y) | 0.883(0.433-1.800) | 0.733 |
| Body Weight (≥64 kg vs. <64 kg) | 1.472(0.859-2.521) | 0.159 |
| BMI (≥24 kg/m^2^ vs. <24 kg/m^2^) | 1.534(0.912-2.581) | 0.107 |
| Diabetes (Yes vs. No) | 2.227(1.125-4.410) | **0.022** |
| Hypertension (Yes vs. No) | 0.766(0.430-1.366) | 0.367 |
| History of other cancer (Yes vs. No) | 1.153(0.160-8.332) | 0.888 |
| Family history of cancer (Yes vs. No) | 1.668(0.819-3.401) | 0.159 |
| History of appendectomy (Yes vs. No) | 0.720(0.288-1.803) | 0.483 |
| Concomitant polyp (Yes vs. No) | 1.007(0.432-2.347) | 0.987 |
| Laparoscopic procedure (Laparoscopic vs. Open) | 0.796(0.377-1.680) | 0.549 |
| Radical resection (Yes vs. No) | 1.597(0.221-11.537) | 0.643 |
| Combined Resection (Yes vs. No) | 0.813(0.198-3.333) | 0.773 |
| Stoma (Yes vs. No) | 1.591(0.882-2.869) | 0.123 |
| Gross Appearance (Protruding vs. Ulcerative) | 1.177(0.673-2.057) | 0.568 |
| Differentiation (Poor vs. Moderately) | 1.049(0.543-2.025) | 0.887 |
| BRAF (Mutant vs. Wild) | 0.821(0.114-5.928) | 0.845 |
| KRAS (Mutant vs. Wild) | 1.127(0.669-1.898) | 0.654 |
| Tumor deposit (Yes vs. No) | 2.576(1.516-4.375) | **<0.001** |
| Vascular invasion (Yes vs. No) | 4.195(2.455-7.170) | **<0.001** |
| Perineural invasion (Yes vs. No) | 0.478(0.205-1.113) | 0.087 |
| Circumferential resection margin | 0.672(0.093-4.856) | 0.694 |
| Distal resection margin | 0.049(0-2847.006) | 0.590 |
| CEA (≥5 ng/mL vs. <5 ng/mL) | 1.160(0.686-1.963) | 0.579 |
| CA199 (≥37 U/mL vs. <37 U/mL) | 1.564(0.767-3.187) | 0.218 |
| Diameter (≥4 cm vs. <4 cm) | 1.191(0.701-2.021) | 0.518 |
| Invasion depth (T3-4 vs. T1-2) | 3.000(1.421-6.336) | **0.004** |
| Lymph node metastasis (N1-2 vs. N0) | 4.266(2.364-7.698) | **<0.001** |
| TNM stage (3-4 vs. 1-2) | 4.266(2.364-7.698) | **<0.001** |
| Postoperative chemotherapy (Yes vs. No) | 0.643(0.405-1.020) | 0.061 |
| Postoperative radiation (Yes vs. No) | 0.515(0.265-1.000) | 0.050 |
