# Supplementary material for: Role of Perirectal Fat in the Carcinogenesis and Development of Early-Onset Rectal Cancer
Source: J Oncol. 2022 Mar 22;2022:4061142. doi: 10.1155/2022/4061142 (PMC8965599; doi:10.1155/2022/4061142)
Supplement: Supplementary 3 — Table S2: univariate analysis for possible risk factors of OS in patients with stage I–III CRC. [file 4061142.f3.docx]

**Table S2.** Univariate analysis for possible risk factors of OS in patients with stage I–III CRC

| **Parameters** | **Odds ratio (95% CI)** | ***P*** |
| --- | --- | --- |
| Perirectal fat area (≥20.2 cm^2^ vs. <20.2 cm^2^) | 1.936(1.038-3.611) | **0.038** |
| Sex (Female vs. Male) | 1.19(0.644-2.201) | 0.578 |
| Age2 (≥50 y vs. <50 y) | 0.637(0.306-1.326) | 0.228 |
| Body Weight (≥64 kg vs. <64 kg) | 1.319(0.719-2.42) | 0.371 |
| BMI (≥24 kg/m^2^ vs. <24 kg/m^2^) | 1.341(0.743-2.422) | 0.330 |
| Diabetes (Yes vs. No) | 1.817(0.81-4.076) | 0.148 |
| Hypertension (Yes vs. No) | 0.579(0.286-1.172) | 0.129 |
| History of other cancer (Yes vs. No) | 0.049(0-1844.424) | 0.574 |
| Family history of cancer (Yes vs. No) | 1.419(0.6-3.357) | 0.426 |
| History of appendectomy (Yes vs. No) | 0.553(0.171-1.786) | 0.322 |
| Concomitant polyp (Yes vs. No) | 0.857(0.306-2.395) | 0.768 |
| Laparoscopic procedure (Laparoscopic vs. Open) | 0.777(0.328-1.838) | 0.566 |
| Radical resection (Yes vs. No) | 1.218(0.168-8.847) | 0.845 |
| Combined Resection (Yes vs. No) | 1.123(0.272-4.641) | 0.872 |
| Stoma (Yes vs. No) | 1.498(0.771-2.908) | 0.233 |
| Gross Appearance (Protruding vs. Ulcerative) | 0.526(0.244-1.131) | 0.100 |
| Differentiation (Poor vs. Moderately) | 0.968(0.45-2.083) | 0.934 |
| BRAF (Mutant vs. Wild) | 0.967(0.133-7.025) | 0.974 |
| KRAS (Mutant vs. Wild) | 1.301(0.721-2.35) | 0.383 |
| Tumor deposit (Yes vs. No) | 3.492(1.932-6.313) | **<0.001** |
| Vascular invasion (Yes vs. No) | 4.824(2.653-8.771) | **<0.001** |
| Perineural invasion (Yes vs. No) | 0.661(0.279-1.564) | 0.346 |
| Circumferential resection margin | 0.869(0.12-6.312) | 0.890 |
| Distal resection margin | 0.049(0-9797.134) | 0.628 |
| CEA (≥5 ng/mL vs. <5 ng/mL) | 1.534(0.849-2.772) | 0.156 |
| CA199 (≥37 U/mL vs. <37 U/mL) | 1.504(0.67-3.374) | 0.322 |
| Diameter (≥4 cm vs. <4 cm) | 0.874(0.484-1.579) | 0.656 |
| Invasion depth (T3-4 vs. T1-2) | 2.576(1.148-5.779) | **0.022** |
| Lymph node metastasis (N1-2 vs. N0) | 5.685(2.731-11.831) | **<0.001** |
| TNM stage (3-4 vs. 1-2) | 5.685(2.731-11.831) | **<0.001** |
| Postoperative chemotherapy (Yes vs. No) | 0.617(0.370-1.028) | 0.064 |
| Postoperative radiation (Yes vs. No) | 0.686(0.349-1.350) | 0.276 |
